# A comparative study of carcass characteristics and meat quality in genetic resources Pekin ducks and commercial crossbreds

**DOI:** 10.5713/ajas.18.0790

**Published:** 2019-02-14

**Authors:** Dariusz Kokoszyński, Dariusz Piwczyński, Henrieta Arpášová, Cyril Hrnčar, Mohamed Saleh, Rafał Wasilewski

**Affiliations:** 1 Department of Animal Sciences, Faculty of Animal Breeding and Biology, UTP University of Science and Technology, Bydgoszcz 85084, Poland; 2 Department of Animal Biotechnology and Genetics, Faculty of Animal Breeding and Biology, UTP University of Science and Technology, Bydgoszcz 85084, Poland; 3 Department of Poultry Science and Small Farm Animals, Faculty of Agrobiology and Food Resources, Slovak University of Agriculture, Nitra 94976, Slovak Republic; 4 Department of Poultry and Animal Production, Faculty of Agriculture, Sohag University, Sohag 82524, Egypt

**Keywords:** Duck, Carcass Traits, Genotype, Meat Quality, Texture, Sex

## Abstract

**Objective:**

The study was aimed to compare carcass traits, physicochemical and textural properties of meat in two different genotypes of Pekin ducks with regard to sex effect.

**Methods:**

The study involved 120 Pekin ducks: 30 males and 30 females of strain P33 (Polish native Pekin ducks) and 30 males and 30 females of Star 53 HY (commercial hybrid Pekin ducks). At 49 d of age, 48 birds (12 males and 12 females of each genotype) were selected for dissection. After the dissection, meat samples were collected to determine meat quality traits.

**Results:**

The studied Pekin ducks of different genotype showed significant differences in body weight, carcass weight, dressing percentage, as well as percentages of breast muscles, skin with subcutaneous fat, abdominal fat, neck, and remainders of eviscerated carcass with neck. Duck genotype influenced the content of crude protein, crude fat, Na, K, P, Zn, pH_24_, electric conductivity (EC_24_), cooking loss, L*, a*, most textural traits of breast muscle, and also Na, Mg and Fe content, EC_24_, drip loss, cooking loss and L*, a*, and b* colour coordinates of leg muscles. Regardless of genetic origin, males exhibited higher BW, carcass weight and carcass neck percentage, as well as lower redness, hardness, chewiness and gumminess of breast muscle compared to females. The genotype×sex interaction was significant for the crude fat content and cooking loss of breast muscle, and for the yellowness of leg muscle.

**Conclusion:**

Star 53 HY ducks are more suited for broiler production due to their higher body weight and dressing percentage. Their breast and leg meat are characterized by more beneficial chemical composition but has poorer sensory and textural properties compared to the meat of P33 ducks.

## INTRODUCTION

Young Pekin fattening ducks are ideal for meeting the requirements of poultry production for meat purposes. They are fast-feathering and exhibit a high growth rate, good feed conversion, proper body conformation, and high dressing percentage. In addition, Pekin ducks are characterized by high viability, relatively low nutritional requirements compared to broiler chickens and young fattening turkeys, high resistance to a harsh rearing environment, and considerable immunity to disease. For the reasons stated above, and due to very good conversion of farm feeds (forages, feed roots, dried feeds, steamed potatoes, silages) and by-product feeds, ducks are highly suitable for backyard and organic farming [1,2].

The interest in rearing young ducks arises mainly from the acquisition of meat that is highly nutritive and has a unique aroma and taste, which makes it suitable for preparation of many exquisite dishes. Duck dishes are particularly popular in French and Chinese cuisine [2]. Good quality feathers and manure are also obtained from fattening ducks.

According to FAO data [3], the world production of duck meat was 4,460,226 tonnes in 2017. Duck meat production accounted for 3.7% of the total poultry meat production, ranking third after chicken and turkey meat production. The largest producer of duck meat was China, which provided 68.8% (3,067,219 tonnes) of global duck meat production. France ranked second with a production of 235,482 tonnes (5.3%), and Myanmar third (3.4%, 152,263 tonnes). Asia accounted for 84.2% (3,754,420 tonnes), Europe for 10.9% (486,024 tonnes), and the other continents for 4.9% of the global duck meat production. In 2017, the proportion of duck meat in poultry meat was 8.5% in Asia, 2.3% in Europe, and as little as 0.3% in North America [3]. Such high disparities are attributable to the different consumer tastes and production costs. Europe and North America show preference for poultry with more meat and less fat, such as broiler chickens and young turkeys. In China and south-east Asia, ducks are mainly consumed by farmers and their fat is a source of energy [4].

In Asia, duck meat is obtained mainly from the slaughter of Pekin ducks. A relatively large proportion is made by the meat from spent ducks. In these regions of the world, ducks are also kept for table eggs, which are highly popular among the local consumers [2]. In north and central Europe, duck meat is obtained principally from Pekin ducks, and in Mediterranean countries (mainly France and Italy) from Muscovy and mallard ducks. In 2017, large producers of duck meat in addition to France were Hungary (52,796 tonnes), Poland (51,243 tonnes), and Germany (35,998 tonnes) [3].

In Poland, duck meat is obtained mainly from young fattening ducks kept under an intensive (farm) system. Two-strain crosses of Pekin ducks form around 90% of the ducks for fattening in Poland.

In a search for new food products that are healthy and safe to eat, consumers from many countries have recently shown renewed interest in the native breeds of waterfowl, including ducks [5]. Based on native poultry breeds, it is possible to produce quality and safe foods desired by consumers in many countries. The carcasses obtained from native ducks aged 8 weeks have a high content of breast muscle (12.9% to 15.8% meat) that is high in protein and low in fat and has a desirable profile of fatty acids rich in polyunsaturated fatty acid, including linoleic and arachidonic fatty acids [6,7]. The findings of Witkiewicz et al [8] show that it is possible to use ducks covered by the genetic resources programme (groups P33, Polish native Pekin ducks; and Mini Duck K2, Polish ducks bred from wild mallards, *Anas platyrhynchos* L., and Pekin ducks) to produce valuable foods, and in breeding programmes aimed to create new strains or to improve the existing ones.

The objective of this study was to compare P33 ducks (Polish native Pekin ducks) covered by genetic resources programme with Star 53 HY ducks (commercial hybrid Pekin ducks) for body weight (BW), carcass weight, dressing percentage, carcass composition, basic chemical composition, content of some minerals, physicochemical and textural characteristics of the meat.

## MATERIALS AND METHODS

### Birds and housing

The study was conducted with 120 Pekin ducks: 30 males and 30 females of strain P33 covered by the genetic resources programme in Poland, as well as 30 males and 30 females of Star 53 HY originating from parents imported from the French Grimaud Fréres company. One-day-old sexed ducks were brought to the farm from a hatchery and divided into groups by genotype. Ducks were kept in a confinement building without outdoor access, on a straw-covered floor, in six pens, each with an area of 12 m^2^. Each genetic group of 60 ducks was divided into three subgroups, each having 10 males and 10 females. Birds from each subgroup were kept in a separate pen located in the middle and each end of the building.

Infrared heaters were used during the first 21 days of rearing. Temperature, relative humidity and air movement were adjusted to bird age. The study was approved by the Local Ethics Committee for Experiments on Vertebrate Animals (no. 21/2014).

### Feeding programme and diets

Throughout rearing (1 to 49 d of age), ducks had *ad libitum* access to water and feed. From 1 to 21 d, they were fed a commercial complete starter diet in crumble form, containing 20.53% crude protein (CP) and 11.85 MJ metabolizable energy (ME)/kg. From 22 to 49 d of rearing, they received a complete pelleted grower/finisher diet containing 17.68% CP and 12.88 MJ ME/kg. The ingredient composition of the starter and grower/finisher diets is given in Table 1. The chemical composition, energy value and content of selected amino acids, calcium, and available phosphorus in the feeds are presented in Table 2.

### Carcass characteristics

All the ducks were individually weighed at 49 d of age with a hook scale Axis BD15S (Axis, Gdańsk, Poland) with an accuracy level of 5 g. Next, 4 males and 4 females whose BWs were similar to the arithmetic mean for a given sex in the pen, were chosen for dissection. A total of 48 birds, 12 males and 12 females of each genotype, were selected for the slaughter. Feed was withdrawn 12 h before slaughter, and the birds were given free access to water. The ducks selected for the dissection were slaughtered, defeathered and eviscerated at the farm of the Department of Animal Sciences, UTP University of Science and Technology in Bydgoszcz. All the ducks selected for the dissection were manually slaughtered. Stunning with a club was followed by cutting the neck blood vessels. During evisceration, the digestive tract and other internal organs were removed from body cavity. Eviscerated carcasses with neck were chilled in a Hendi refrigerated chamber (Hendi, Gądki, Poland) at 4°C for 18 h. Chilled carcasses were weighed on a WLC 6/12/F1/R electronic balance (Radwag, Radom, Poland), accurate to 0.1 g.

Next, the whole eviscerated carcasses were dissected according to the method described by Ziołecki and Doruchowski [9]. Each eviscerated carcass was dissected into: neck without skin, wings with skin, breast muscles (pectoralis major muscle plus pectoralis minor muscle), leg muscles (all thigh and drumstick muscles), abdominal fat, skin with subcutaneous fat from the whole carcass, without skin from the wings, and the remainder of the carcass. The remainder of the carcasses was formed by the skeleton with a certain amount of skeletal muscles (intercostal, dorsal, suprascapular, and other) together with kidneys, but without lungs and other internal organs. The dissected carcass components were weighed accurate to 0.1 g on the WLC 6/12/F1/R electronic balance. The data obtained were used to calculate their percentage in eviscerated carcass with neck. The data on the weight of eviscerated carcass with neck after chilling and the BW of the ducks selected for slaughter, were used to calculate performance of the ducks from the compared groups, with regard to sex.

### Meat quality

Before the carcasses were cut 24 h after slaughter, the pH of breast muscles (pectoralis major muscle) and leg muscles (drumstick muscle) was measured with a pH Star CPU meter (Ingenieurbüro R. Matthäus, Nobitz, Germany) equipped with a combination glass electrode for determination of meat pH. pH values were determined with an accuracy of 0.01. Prior to the measurements, the pH meter was calibrated with standard solution (pH 7.0 and 5.5) and adjusted to the meat temperature (4°C). Electric conductivity (mS/cm) was measured with an LF-Star CPU device (Ingenieurbüro R. Matthäus, Germany). The device’s stainless-steel electrodes were placed parallel with the breast muscle fibres at an angle of 90°. The measurement was accurate to 0.1 mS/cm.

After the dissection, the breast and leg muscles were individually sampled from each carcass to determine basic chemical composition, the content of seven minerals, meat colour coordinates, drip loss and cooking loss. The pectoralis major muscles were also sampled to determine their texture.

The basic chemical composition of the dissected breast and leg muscles from the ducks of the compared genetic groups, was determined using standard methods (AOAC International) at the Laboratory of Feed and Animal Raw Materials Quality of the Faculty of Animal Breeding and Biology, UTP University of Science and Technology in Bydgoszcz, Poland. The amount of protein (N×6.25) was determined by Kjeldahl using 2200 Kjeltec Auto Distillation (Foss Tecator AB, Höganäs, Sweden). The fat content of the analysed meat samples was determined with a Soxtec System HT 1043 Extraction Unit (Foss Tecator AB, Sweden), and the amount of water in the samples by the drying method.

The content of sodium (Na), potassium (K), magnesium (Mg), zinc (Zn), iron (Fe), copper (Cu), and phosphorus (P) was determined at the Laboratory of Feed and Animal Raw Materials Quality of the Faculty of Animal Breeding and Biology, UTP University in Bydgoszcz. To this end, breast and leg meat samples were lyophilized in an Alpha 1–4 LD plus freeze-drier (Christ, Ostende, Germany) and wet mineralized in an Ethos Plus microwave digester (Milestone, Sorisole, Italy). The analysis of Na, K, Mg, Zn, Fe, and Cu was performed by flame atomic absorption spectroscopy using a Solar 969 spectrometer (Unicam, Cambridge, UK). Phosphorus was determined with a Marcel Media Eko spectrophotometer (Marcel, Warsaw, Poland). The samples were prepared and determined in accordance with Polish standards.

Drip loss and cooking loss of the meat were also measured. To determine the cooking loss of the breast and leg meat samples weighing 20±2 g, were ball-shaped, wrapped in an absorbent cheesecloth and placed in a water bath at 85°C for 10 min [10]. After removing from the water bath, the samples were chilled for 30 min in a refrigerating cabinet (Hendi, Poland) at 4°C and again weighed on a PS 750/X balance (Radwag, Poland) with an accuracy of 0.01 g. Cooking loss was calculated as a difference in the weight of raw and heat-treated meat samples, and expressed in percent of the initial sample.

Drip loss was determined on the samples of pectoralis major muscle and leg (thigh and drumstick) muscles. Each muscle was weighed on the above balance to the nearest 0.01 g, placed separately in a bag perforated on the bottom to allow meat juice to drain away, and then placed in a second bag to prevent contact between the draining juice and the meat sample. The samples were suspended on racks and stored at 4°C for 24 h in a Hendi refrigerating cabinet (Hendi, Poland). After that time, the meat samples were reweighed. Drip loss was calculated in percent from the difference in weight before and after chilling.

The colour of meat was determined according to the Commission Internationale de l’Eclairage (CIE) system [11] on the inner surface of raw breast muscles (pectoralis major muscle) and leg muscles (thigh and drumstick muscles, after removing the patella and tendons, and following grinding) obtained after cutting up of the carcasses. The parameters L* (colour lightness), a* (relative redness [on red-green axis]), and b* (relative yellowness [on yellow-blue axis]) were measured using a model CR410 colorimeter (Konica Minolta, Tokyo, Japan). The measurement area was 50 mm in diameter. The chroma meter was calibrated against a CR410 white reference tile (Y = 94.40, x = 0.3159, y = 0.3325). The determinations were made at the Department of Animal Sciences of the UTP University of Science and Technology in Bydgoszcz.

The meat texture (hardness, cohesiveness, springiness, chewiness, gumminess, Warner-Bratzler [WB] shear force) was tested on the heat-treated pectoralis major muscle at the Department of Meat Technology of the West Pomeranian University of Technology in Szczecin, Poland. The meat texture was determined using texture profile analysis (TPA) and WB tests with an Instron 1140 apparatus (Instron Corp., Norwood, MA, USA).

The meat samples were packed into foil bags, heated in water at 72°C±2°C until the temperature reached 70.2°C in the geometric centre, cooled in cold water until reaching 20°C, packed into food packaging film, stored for 12 h in the refrigerator at 4°C, and heated to 18°C. After removing the film, 20±1 mm thick slices were cut out from the samples to determine the meat texture.

TPA test involved a double immersion of the plunger 0.96 cm in diameter and 20±1 mm high, at the depth of 16 mm. Based on the curve representing the strength-deformation dependence, the following parameters were determined: hardness, the maximum height of peak I; cohesiveness, the ratio of the area of peak II to the area of peak I, springiness of the base of the rising part of peak II; gumminess, the product of hardness and cohesiveness; chewiness, the product of hardness, cohesiveness and springiness [12].

The WB test involved cutting the sample, across the muscle fibres, with the cross-section 10×10 mm, using a triangle knife. Each sample was determined 5 times (5 replications), and the average for each sample was calculated. The working speed of the crosshead for the test was 50 mm/min. The test allowed for determining WB shear force.

### Statistical analysis

The numerical data on the BW, weight of the carcass and carcass cuts, dressing percentage, basic chemical composition, the content of some minerals, as well as physicochemical and textural traits, were statistically analysed. Arithmetic means and standard error of mean were calculated for each trait (for both groups together). The two-factor analysis of variance was used to examine the effect of genotype and sex on the above meat traits of the ducks. To this end, the following linear model was applied: y_ijk_ = μ+a_i_+b_j_+(a×b)_ij_+e_ijk_, where y_ijk_ is the value of the analysed trait, μ is the overall mean of the analysed trait, a_i_ is the effect of i-th duck genotype, b_j_ is the effect of j-th sex, (a×b)_ij_ is the genotype by sex interaction, and e_ijk_ is random error.

Statistical analyses of the BW, weight of the carcass and carcass cuts, dressing percentage and meat quality traits were made with the use of SAS software, version 9.4 [13]. Significant differences between the compared groups and between the males and females were determined by the Tukey test. The level of significance was at p<0.05. The individual bird was the experimental unit for all the analysed traits.

## RESULTS

### Slaughter traits

The compared groups of Pekin ducks differed (p<0.05) in the mean BW, slaughter weight, carcass weight, and dressing percentage. The commercial Pekin Star 53 HY hybrids had significantly higher values of these traits compared to the Polish Pekin of strain P33. Regardless of genotype, males exhibited significantly (p<0.05) higher BW and carcass weight. Sex had no significant effect on the dressing percentage of ducks aged 49 d. The carcasses obtained from 49-d-old Star 53 HY ducks had significantly higher percentage of breast muscles and the remainder of the carcass, as well as significantly lower content (%) of skin with subcutaneous fat and abdominal fat, and of neck compared to P33 ducks of the same age. Compared to females, males had a significantly (p<0.05) higher proportion of neck in the carcass. The genotype×sex interactions for the evaluated slaughter traits were not significant (Table 3).

### Chemical composition

Star 53 HY and P33 ducks did not differ in the basic chemical composition except for the CP and crude fat content of breast muscle. Significantly higher CP content was determined in the breast muscles of commercial Pekin Star 53 HY hybrids than in Polish native Pekin ducks of strain P33. Polish Pekin ducks had a significantly higher content of crude fat in breast muscle. An inverse pattern was found for leg muscles. Sex of birds did not have a significant effect on the dry matter, CP and crude fat content of breast and leg muscles in 49-d-old Pekin ducks of different genotypes. An interaction between genotype and sex (p = 0.038) was only detected in the crude fat content in breast muscle (Table 4).

At the age of 49 d, Polish Pekin ducks (strain P33) had a significantly (p<0.05) higher content of sodium and phosphorus as well as a significantly lower content of potassium and zinc in breast muscles compared to the commercial Star 53 HY ducks. The leg muscles of Polish Pekin ducks (strain P33) contained significantly less sodium, magnesium and iron compared to the leg muscles of commercial Pekin duck hybrids. Regardless of genotype, there were no significant (p<0.05) differences between males and females in the mineral content (Na, K, P, Mg, Zn, Fe, and Cu) of breast and leg muscles from the compared genetic groups of Pekin ducks aged 49 d. The genotype×sex interactions for the content of these minerals were not significant (Table 5).

### Meat physicochemical properties

The breast muscles of P33 ducks exhibited significantly lower pH_24_, electric conductivity (EC_24_), cooking loss, and redness (a*) as well as significantly higher lightness (L*) compared to Star 53 HY hybrids. For the leg muscles, significantly lower EC_24_, drip loss, cooking loss, redness (a*) and significantly higher lightness (L*) and yellowness (b*) were observed in P33 ducks compared to Star 53 HY hybrids. An interaction between genotype and sex was only found for the cooking loss of breast muscles (p = 0.014) and for the yellowness (b*) of leg muscles (p = 0.011) (Table 6).

### Meat texture

In 49-d-old ducks, genotype significantly affected hardness, chewiness, gumminess and WB shear force. The heat-treated pectoralis major muscle of P33 ducks showed significantly (p<0.05) lower hardness, chewiness, gumminess, and WB shear force compared to the breast muscle of Star 53 HY ducks. Regardless of duck genotype, the heat-treated pectoralis major muscle of males had lower (p<0.05) hardness, chewiness, gumminess compared to female breast muscles. The genotype ×sex interactions for the analysed textural traits of the pectoralis major muscle were not significant (Table 7).

## DISCUSSION

The Pekin ducks of different genotype, which were evaluated in this experiment, showed differences in BW at 49 d of age. P33 ducks (Polish native Pekin ducks) are one of the 10 flocks included in the genetic resources conservation programme in Poland. Birds in these flocks are selected only for conformation and health, but not for productive traits. The large differences in BW at 49 d of age are due to the lack of selection for this trait in P33 ducks, unlike Star 53 HY hybrids. Książkiewicz and Kiełczewski [14], who analysed the results for meat traits in 8 generations of Pekin ducks, including strain P33, found higher BW in 49-d-old males (2,492 g) and females (2,405 g) in comparison to our study. In the study by Witkiewicz et al [8], the BW of P33 ducks aged 49 d averaged 3,050 g for males and 2,670 g for females. The mean BW of the 49-d-old P33 ducks in our study was similar to the BW of ducks of the same origin and age [15], which were studied in 2015 at the Waterfowl Genetic Resources Station in Dworzyska, belonging to the National Research Institute of Animal Production in Kraków, as part of the official performance testing of duck flocks included in the genetic resources conservation programme. In the experiment by Kokoszyński et al [4], the BW of 49-d-old Star 53 HY hybrids was 3,469 g in males and 3,186 g in females, being higher when compared to the ducks from our study. Biesiada-Drzazga et al [16] reported the mean BW of Star 53 HY ducks aged 49 d to be 2,859 g in males and 2,641 g in females.

The compared groups of ducks of different genotype were characterized by high dressing percentage. However, the ratio of the weight of eviscerated carcass with neck to the slaughter weight of the ducks aged 49 d in our study was lower than in Pekin ducks of the same age studied by Hong et al [17], and in Pekin ducks aged 45 d in the experiment of Chae et al [18]. Lower dressing percentage than in the ducks evaluated in our study at the age of 49 d was observed in Pekin ducks by Witak [19]. In our study, Star 53 HY hybrids showed higher dressing percentage compared to P33 ducks, which supports the finding of Murawska [20] that ducks with higher BW have higher dressing percentage due to the lower percentage of offals (inedible guts, head, feet) and giblets.

The dissection analysis showed that the compared drakes and ducks of different genotype differed significantly in percentage of breast muscles in eviscerated carcass with neck. Kisiel [21] reported that the proportion of breast muscles in the carcasses of 49-d-old P33 ducks was 8.2% in males and 10.1% in females. A higher proportion of breast muscles in P33 ducks aged 49 d (males 13.0%, females 12.6%) was noted by Witkiewicz et al [8]. Gornowicz and Szukalski [22], in 56-d-old P33 ducks, found higher breast muscle percentage in eviscerated carcass with neck, without giblets (15.2% in males, 16.2% in females) than in 49-d-old ducks from our study. This may suggest that 56-d-old P33 ducks are more useful for the consumer compared to those aged 49 d. The weak development of breast muscles in the evaluated P33 ducks was related mainly to the lack of selection carried out in the ducks of this strain, including the improvement of breast muscling. In our study, the carcasses of P33 females had a higher percentage of breast muscles than male carcasses, which is consistent with the results obtained for the genetic reserve ducks [22]. On the other hand, Witak [19] found higher breast muscle percentages in male than female carcass, the same as with Star 53 HY ducks in our study. The differences obtained in the breast muscle content of carcasses are probably due to the different growth rates of the ducks under study. The proportion of skin with subcutaneous fat in eviscerated carcasses from 49-d-old P33 ducks exceeded 26% and was similar to the earlier results for ducks of the same genotype [14]. The obtained results confirm the high fatness of the genetic reserve Pekin ducks. The evaluated P33 ducks also had a high proportion of abdominal fat, which was higher than in 49-d-old Pekin ducks investigated by Kisiel [21]. A higher abdominal fat content than in our study was reported for genetic reserve Pekin ducks by Witkiewicz et al [8]. However, most subcutaneous fat is rendered out during heat treatment, while abdominal fat can be removed before heat treatment, which reduces the amount of fat (and the amount of energy) eaten by the consumers of duck meat.

The compared ducks of different genotypes had a high content of CP in breast and leg muscles. Higher CP content was determined in breast than in leg muscles. In the study by Mazanowski and Bernacki [23], the content of CP in 49-d-old breeding ducks ranged from 18.8% (strain K11) to 19.5% (strain P66) in breast muscles, and from 17.6% (strain P77) to 18.7% (strain P66) in leg muscles, which is less than in our study. Muhlisin et al [7] found significantly higher protein content in the breast muscles of Korean native ducks than in imported commercial ducks. In turn, Hong et al [17] found significantly higher protein content in breast muscles of strain B compared to strain A of Korean native ducks aged 49 d as well as no significant differences in the amount of protein in breast muscles of ducks aged 42 and 56 d. Heo et al [24], in two-strain hybrids (AA, AB, BB, BA) of Korean native duck found no significant differences in the protein content of breast muscles. Muhlisin et al [7] found significantly higher fat content in breast muscles of imported commercial ducks compared to Korean native ducks. In another experiment [24], genotype had no significant effect on fat content in breast muscles of ducks of the same age.

Our experiment also determined the content of some minerals, namely sodium, potassium, phosphorus, magnesium, zinc, iron and copper in breast and leg muscles. Apart from playing important physiological functions, minerals contribute to the meat quality traits such as colour, acidity, water holding capacity, and taste. Połtowicz and Doktor [25] observed positive effects of magnesium on pH_15_ and drip loss, of phosphorus on pH_15_, and of potassium on water holding capacity of poultry meat.

The mineral content of the meat from the experimental ducks was affected by genotype. Kokoszyński et al [4] observed significant differences in the sodium, potassium, phosphorus and iron content of leg muscles in SM3 Heavy, Star 53 HY and AF51 hybrids of Pekin ducks. The leg muscles of Star 53 HY ducks of both sexes had a significantly higher sodium and iron content compared to SM3 Heavy and AF51 ducks. In the leg muscles of SM3 Heavy ducks of both sexes, there was significantly more potassium and phosphorus compared to Star 53 HY hybrids. In our study we found significantly higher iron content in leg muscles of Star 53 HY compared to ducks of strain P33, which contributed to a significantly darker colour (lower L*) of the leg muscles from Star 53HY compared to P33 ducks. These results confirm the relationship between iron concentration and meat colour, which was reported in an earlier study [26].

pH is an important indicator of meat quality. In our study, pH of breast muscles was significantly higher in Star 53 HY ducks than in P33 ducks when measured 24 h postmortem. The pH_24_ values of breast muscles from the analysed Pekin ducks were lower than those in the breast muscles of ducks reported by Kokoszyński et al [27]. Similar pH_24_ values of breast muscles as in the ducks from our study were obtained by Erdem et al [28]. Uhlirová et al [5] reported that differences in the pH values result from the differences in the glycogen reserves at slaughter, response to preslaughter stress or slaughter weight.

Another, relatively little-known parameter of duck meat quality is its electrical conductivity. Normal quality (pale, soft, exudative; and dark, firm, dry free) meat has low electrical conductivity measured postmortem, which increases with storage time. Kokoszyński et al [27], similar to our study, found high EC_24_ values for breast muscles (control 6.99 mS/cm, experimental 8.12 mS/cm) and leg muscles (control 6.49 mS/cm, experimental 9.24 mS/cm) in 49-d-old SM3 Heavy ducks.

In our study, we also determined drip loss and cooking loss, which showed a significant effect of genotype on drip loss of leg muscles and of cooking loss on drip loss of breast and leg muscles from the studied Pekin ducks aged 49 d. Muhlisin et al [7] found lower cooking loss from breast muscle of Korean native ducks and imported commercial ducks compared to Star 53 HY ducks from our study, and higher cooking loss than in P33 ducks. The same authors also observed that genotype has a significant effect on the cooking loss of breast muscle. Heo et al [24] found significantly higher cooking loss in BA than in AA, AB, and BB hybrids of Korean native ducks, whereas Onk et al [29] noted higher cooking loss in pectoralis major muscle from native ducks than in the same muscle from Pekin ducks. Chartrin et al [30] observed significantly higher cooking loss of breast muscle from Pekin ducks compared to mule, Muscovy and hinny ducks, while Wołoszyn et al [31] found significantly higher cooking loss in breast muscles of Khaki Campbell ducks compared to Pekin and Mini Duck (hybrid of wild mallard duck *Anas platyrhynchos* and Pekin duck). In the experiment by Ali et al [32], drip loss from breast muscles of 48-d-old ducks ranged from 1.7% to 1.81%, which is similar or higher than in our study.

Another trait of importance to consumers and processors is the colour of meat. In the commercial evaluation of meat quality, it is indicative of freshness and suitability for specific culinary purposes, and it is strictly associated with the ultimate pH. This trait serves an important role in the production of meat preparations, which should have a clear and stable colour in the cross-section [33]. In our study, we observed a significant effect of genotype on the lightness (L*) and redness (a*) of breast muscles and on all colour coordinates (L*, a*, b*) of leg muscles. Muhlisin et al [7] found significantly higher L* (lightness) and b* (yellowness) values of breast muscles from imported commercial ducks compared to Korean native ducks, which partially disagrees with our findings. The same authors observed higher L* and significantly higher a* and b* values in the leg muscles of Korean native duck than in imported commercial ducks from Grimaud Fréres company. Gornowicz et al [33] did not observe any significant effect of sex on the L*, a*, and b* values of breast and leg muscles from Pekin ducks, which is consistent with our study apart from the a* value of breast muscles. In our study, the higher L* values of breast muscles in P33 compared to Star 53 HY ducks probably result from the higher amount of fat in the muscles of genetic reserve ducks and from the higher stress susceptibility of the heavier Star 53 HY ducks. This had an adverse effect on the exsanguination of the ducks, and thus increased the haemoglobin content of meat, which influences meat colour in addition to myoglobin and its derivatives. The darker colour of breast muscles from Star 53 HY ducks was associated with the higher pH_24_ value, which may suggest their higher sensitivity to the rearing house environment.

In our experiment, higher breast muscle tenderness was found in P33 than in Star 53 HY ducks, which is probably associated with the lower muscle fibre diameter of lighter P33 ducks compared to Star 53 hybrids. Witkiewicz et al [8] found a significantly smaller diameter of white and red fibres and a significantly higher percentage of red fibres in unselected P33 and K2 ducks from genetic reserve flocks than in selected pedigree ducks of strains A44 and P66. In the study by Lee et al [34], breast muscle tenderness was higher in male than in female Korean native ducks and commercial ducks. Chartrin et al [30] observed higher tenderness of breast muscles with a higher lipid content (from 3.06% to 4.37% and above) when compared with breast muscles of the ducks with a lower lipid content (from 1.7% to 3.05%).

## CONCLUSION

In summary, the Star 53 HY hybrid ducks aged 49 d exhibited significantly higher BW, carcass weight and dressing percentage compared to P33 birds of the same age. Eviscerated carcasses from 49-d-old Star 53 HY ducks better meet consumer expectations due to the higher meat percentage (including a significantly higher breast muscles percentage) and significantly lower fatness (percentage of skin with subcutaneous fat and abdominal fat) compared to P33 ducks. The breast meat of Star 53 HY ducks, due to the high protein and zinc content and low fat and sodium content, could be useful in following the optimum diet and maintaining human health. However, the lower acidity, dark colour, high electrical conductivity, high cooking loss and drip loss, and significantly higher hardness, chewiness, gumminess and WB shear force values of the meat from Star 53 HY ducks is indicative of its lower technological suitability and poorer sensory attributes compared to the meat from P33 ducks.

## Figures and Tables

**Table 1:** Ingredients of the diets for ducks

Ingredients (g/kg as fed)	Starter1 to 21 d	Grower/finisher22 to 49 d
Corn	367.0	438.0
Wheat, ground	175.0	250.0
Wheat meal	40.0	25.0
Wheat middlings	28.0	-
Triticale, ground	50.0	-
Soybean meal (485 g CP/kg)	237.0	173.0
Sunflower seed meal (380 g CP/kg)	25.0	20.0
Rapeseed meal (357 g CP/kg)	40.0	10.0
Corn DDGS (280 g CP/kg)	-	30.0
Soybean oil	5.0	26.0
Limesterine	9.8	9.8
Monocalcium phosphate	10.7	7.4
Sodium chloride	2.13	2.37
Sodium bicarbonate	2.2	1.4
DL-methionine	1.2	1.05
L-lysine	1.27	0.98
Threonine	0.7	-
Vitamin-mineral premix1)	5.0	5.0

CP, crude protein; DDGS, dried distillers grains with solubles.

1) 1 kg of vitamin-mineral premix provided: retinol 10,000 IU, cholecalciferol 2,500 IU, α-tocopherol 20.00 mg, thiamine 0.5 mg, riboflavin 5.00 mg, niacinamide 20.00 mg, pyridoxine 1.0 mg, cobalamine 0.02 mg, folic acid 0.5 mg, menadione 2.5 mg, choline chloride 200.00 mg, Fe 45.00 mg, Mn 62.5 mg, Zn 50.00 mg, Cu 5.00 mg, Se 0.25 mg, I 1.3 mg.

**Table 2:** Chemical composition of the diets for ducks

Ingredients (g/kg as fed)	Starter1 to 21 d	Grower/finisher22 to 49 d
DM (%)	90.46	90.81
CP (g/kg as fed)	205.3	176.8
Crude fat (g/kg as fed)	32.2	40.0
Crude fibre (g/kg as fed)	39.0	51.7
Crude ash (g/kg as fed)	52.0	51.6
N-free extracts (g/kg as fed)	576.1	588.0
ME1) (MJ/kg of fed)	11.85	12.88
Calculated composition (g/kg as fed)		
Lysine	11.3	8.9
Methionine	4.6	4.1
Threonine	8.0	6.2
Tryptophan	2.6	2.1
Calcium	9.1	8.2
Phosphorus soluble	6.5	5.2

DM, dry matter; CP, crude protein; ME, metabolizable energy.

1) The values are calculated from ingredient apparent metabolisable energy values.

**Table 3:** Body weight and carcass characteristics of 49-d-old Pekin ducks of different genotypes

Trait	Genotype (G) – sex (S)				SEM	p-value		

	P33 strain (Polish native Pekin ducks)		Star 53 HY (Commercial hybrid Pekin ducks)					
		
	Male	Female	Male	Female		G	S	G×S
BW, all birds (g)	2,044	1,943	3,126	3,081	83.4	<0.001	0.006	0.879
BW, birds selected for dissection (g)	2,065	1,967	3,162	3,062	81.8	<0.001	0.003	0.974
Carcass weight (g)	1,373	1,357	2,164	2,079	67.8	<0.001	0.039	0.126
Dressing percentage (%)	66.5	66.9	68.4	67.9	0.7	<0.001	0.364	0,155
Breast muscles (%)	10.0	10.2	17.1	16.3	0.5	<0.001	0.102	0.872
Leg muscles (%)	11.4	11.1	12.1	13.4	0.3	0.075	0.088	0.081
Skin with subcutaneous fat (%)	26.7	28.5	19.6	18.9	1.0	<0.001	0.230	0.546
Abdominal fat (%)	1.4	1.5	1.1	0.8	0.1	0.010	0.488	0.232
Neck (%)	10.1	9.3	8.3	7.7	0.2	<0.001	0.028	0.769
Wings (%)	13.6	12.8	13.8	13.8	0.3	0.053	0.645	0.691
Remainders (%)	26.8	26.6	28.0	29.1	0.7	0.002	0.436	0.255

BW, body weight; SEM, standard error of the mean.

**Table 4:** Basic chemical composition of breast and leg meat from 49-d-old Pekin ducks of different genotypes

Trait		Genotype (G) – sex (S)				SEM	p-value		

		P33 strain (Polish native Pekin ducks)		Star 53 HY (Commercial hybrid Pekin ducks)					
		
		Male	Female	Male	Female		G	S	G×S
Dry matter (%)	BM	27.1	26.4	27.0	26.6	0.2	0.897	0.344	0.769
	LM	29.3	28.8	28.6	29.8	0.3	0.819	0.682	0.285
Crude protein	BM	21.7	21.4	23.2	23.4	0.2	<0.001	0.056	0.942
	LM	20.5	21.4	20.2	20.9	0.2	0.328	0.054	0.831
Crude fat	BM	3.1	2.9	1.6	1.6	0.2	0.028	0.809	0.038
	LM	6.7	5.1	6.1	7.3	0.4	0.405	0.879	0.155

SEM, standard error of the mean; BM, breast muscles; LM, leg muscles.

**Table 5:** Content of some minerals in mg/100 g of breast and leg meat from 49-d-old Pekin ducks of different genotypes

Trait		Genotype (G) – sex (S)				SEM	p-value		

		P33 strain (Polish native Pekin ducks)		Star 53 HY (Commercial hybrid Pekin ducks)					
		
		Male	Female	Male	Female		G	S	G×S
Na, sodium	BM	106.7	112.6	82.7	85.9	2.9	<0.001	0.344	0.782
	LM	87.3	84.3	97.8	97.7	1.5	0.004	0.588	0.615
K, potassium	BM	343.8	370.9	375.0	385.7	4.5	0.023	0.056	0.390
	LM	310.5	320.7	334.5	327.8	3.8	0.093	0.846	0.351
P, phosphorus	BM	47.0	48.0	45.4	42.6	0.7	0.042	0.570	0.235
	LM	38.7	40.5	40.0	38.4	0.4	0.734	0.953	0.110
Mg, magnesium	BM	23.7	23.5	24.8	23.6	0.3	0.448	0.396	0.547
	LM	19.4	20.3	22.5	21.5	0.3	0.002	0.969	0.086
Zn, zinc	BM	1.2	1.3	1.4	1.6	0.1	0.007	0.051	0.194
	LM	2.8	2.9	3.2	2.9	0.1	0.124	0.652	0.121
Fe, iron	BM	3.4	3.3	3.6	3.9	0.1	0.240	0.629	0.615
	LM	1.4	1.5	3.0	2.8	0.1	<0.001	0.779	0.355
Cu, copper	BM	0.4	0.4	0.4	0.4	0.1	0.367	0.986	0.957
	LM	0.2	0.2	0.2	0.2	0.1	0.583	0.583	0.581

SEM, standard error of the mean; BM, breast muscles; LM, leg muscles.

**Table 6:** Some physicochemical traits of breast and leg meat from 49-d-old Pekin ducks of different genotypes

Trait		Genotype (G) – sex (S)				SEM	p-value		

		P33 strain (Polish native Pekin ducks)		Star 53 HY (Commercial hybrid Pekin ducks)					
		
		Male	Female	Male	Female		G	S	G×S
pH_24_	BM	5.65	5.70	5.92	5.95	0.1	0.001	0.152	0.777
	LM	6.28	6.46	6.30	6.54	0.1	0.418	0.064	0.254
EC_24_ (mS/cm)	BM	7.9	8.1	9.2	9.1	0.2	0.012	0.848	0.701
	LM	8.2	8.3	9.2	9.5	0.2	0.017	0.619	0.878
Drip loss (%)	BM	1.2	1.4	1.7	1.9	0.1	0.129	0.912	0.448
	LM	0.6	0.7	1.1	1.1	0.1	0.005	0.869	0.981
Cooking loss (%)	BM	19.2	14.9	35.1	36.8	1.7	<0.001	0.257	0.014
	LM	28.7	31.2	36.5	39.3	1.6	0.005	0.869	0.981
L*, lightness	BM	48.1	49.9	43.9	43.7	0.7	0.002	0.582	0.497
	LM	52.3	50.8	43.0	43.2	0.3	0.002	0.728	0.656
a*, redness	BM	17.3	18.3	18.6	20.6	0.3	0.017	0.049	0.514
	LM	14.4	16.0	18.4	17.9	0.9	0.006	0.957	0.200
b*, yellowness	BM	6.6	6.6	6.5	7.1	0.3	0.779	0.663	0.648
	LM	5.3	6.5	5.7	4.0	0.5	0.028	0.414	0.011

SEM, standard error of the mean; BM, breast muscles; LM, leg muscles; EC_24_, electric conductivity.

**Table 7:** Textural traits of pectoralis major muscle from 49-d-old Pekin ducks of different genotypes

Trait	Genotype (G) – sex (S)				SEM	p-value		

	P33 strain (Polish native Pekin ducks)		Star 53 HY (Commercial hybrid Pekin ducks)					
		
	Male	Female	Male	Female		G	S	G×S
Hardness (N)	28.7	31.3	34.5	39.2	1.0	<0.001	0.033	0.927
Cohesiveness	0.4	0.4	0.4	0.4	0.1	0.337	0.748	0.630
Springiness (cm)	1.2	1.1	1.1	1.1	0.2	0.128	0.836	0.143
Chewiness (N×cm)	12.1	14.3	14.6	18.2	0.5	0.003	0.006	0.517
Gumminess (N)	8.6	11.5	14.2	15.4	0.6	0.001	0.049	0.428
Warner-Bratzler shear force (N)	68.4	64.9	76.2	73.3	1.6	0.007	0.834	0.540

SEM, standard error of the mean.
